# Investigation of Eating Habits in Patients with Functional Dyspepsia

**DOI:** 10.5152/tjg.2022.21502

**Published:** 2022-08-01

**Authors:** Hatice Çolak, Fatma Esra Güneş, Yeşim Özen Alahdab, Berna Karakoyun

**Affiliations:** 1 Department of Nutrition and Dietetics, Üsküdar University Faculty of Health Sciences, İstanbul, Turkey; 2 Department of Nutrition and Dietetics, İstanbul Medeniyet University Faculty of Health Sciences, İstanbul, Turkey; 3 Department of Internal Diseases, Marmara University Faculty of Medicine, İstanbul, Turkey; 4 Department of Physiology, University of Health Sciences Hamidiye Faculty of Medicine, İstanbul, Turkey

**Keywords:** Abdominal pain, dyspepsia, habit, nutrition, obesity

## Abstract

**Background:**

Nutritional habits of patients with functional dyspepsia can affect the progression of functional dyspepsia. We aimed to determine the foods and dietary habits that may cause symptoms of postprandial fullness, early satiety, epigastric pain, and epigastric burning in functional dyspepsia patients.

**Methods:**

Sixty functional dyspepsia patients, who were diagnosed according to Rome IV criteria in the endoscopy unit of a gastroenterology institute, were included in the study. Data on the demographic characteristics, anthropometric measurements, nutritional habits, and food consumption frequency questionnaire of functional dyspepsia patients were collected.

**Results:**

Postprandial fullness was found more common in those who preferred roasting as a cooking method. There was no significant difference between symptoms and meal frequency. Epigastric burning and pain were found to be more pronounced in women, and alcohol consumption was less in patients who experienced more epigastric pain. In non-smoker participants, the complaint of early satiety was lower. It was found that broccoli, radish, celery, green olives, and olive oil consumption was less in participants who experienced excessive postprandial fullness. Patients with stomach pain consumed less dry fruits, green olives, butter, alcohol, and fast food. It was found that patients with stomach burning consumed less alcohol and fast food.

**Conclusion:**

In conclusion, functional dyspepsia patients should avoid or reduce consuming broccoli, radish, celery, green olives, olive oil, dry fruits, and butter which may trigger symptoms. Reducing consumption of these foods, abandoning unhealthy cooking methods such as roasting, reducing smoking, and reducing consumption of alcohol and fast food might be beneficial for relieving symptoms.

## Main Points

- Functional dyspepsia (FD) symptoms are affected by the cooking methods, and roasting can trigger postprandial fullness.
- Functional dyspepsia symptoms are influenced by gender, alcohol use, and smoking.
- It has been observed that patients with FD may avoid or rarely consume some foods that may trigger symptoms, either intentionally or unintentionally.
- Healthy eating habits and avoiding foods that trigger symptoms may be beneficial for relieving FD symptoms and improving quality of life.

## Introduction

Functional dyspepsia (FD) is a gastrointestinal disorder with an unknown etiology and varying pathophysiology. It is localized in the epigastric region with pain or discomfort and characterized by persistent or recurrent upper gastrointestinal symptoms. Clinical findings include gastrointestinal symptoms such as inability to finish a normal meal, epigastric fullness, early satiety, pain or discomfort, epigastric burning, postprandial fullness, nausea, itching, and vomiting.^1^ The prevalence of FD ranges from 5% to 29% worldwide and is estimated to be 20%-25% in Turkey.^2,3^ Since the etiology of FD is unknown and it has variable pathogenesis, no definitive treatment method can be offered. The effectiveness of the recommended drug therapies is temporary. Functional dyspepsia symptoms are observed to recur for many years in patients. There are also opinions that uncontrolled and recurrent symptoms may cause cancer in the future.^4^

Dyspeptic symptoms of individuals may be related to food consumption. It has been concluded that the possible mechanisms of dyspeptic symptoms caused by foods include abnormal gastric motor responses during the digestion of food, postprandial fullness, acid hypersensitivity, duodenal hypersensitivity, food allergy, food intolerance, or sensitivity.^5,6^ High-fat foods can cause symptoms such as postprandial fullness, nausea, and satiety by stimulating plasma cholecystokinin levels in patients with FD.^7^ Sensitivity to coffee and acidic foods also increases in FD. There is evidence that coffee and citrus fruits, such as oranges, stimulate the gastrin hormone secretion by increasing gastric acidity and triggering symptoms.^8^ It is known that patients with FD can usually tolerate small amounts in 1 meal. Duncanson et al.^9^ found that women with FD receive lower energy, carbohydrate, protein, fat, and vitamin C when compared to the control group.

Dyspeptic symptoms can also be affected by many eating behaviors, such as the frequency of main and snacks, portion sizes, fluid consumption, eating fast and without chewing.^10,11^ It has been shown that behavioral changes related to eating habits in patients with FD reduce or eliminate the symptoms. Studies have shown that healthy eating habits improve clinical symptoms such as early satiety, epigastric pain, postprandial fullness, and epigastric burning. However, different foods or several eating habits were examined in each of these studies.^5,10,11^

The nutritional habits of patients with FD can affect the frequency of symptoms such as postprandial fullness, early satiety, epigastric pain, and epigastric burning. However, it is still unclear which eating habits trigger or improve these symptoms. We hypothesize that changing eating habits and avoiding foods that may trigger symptoms would be beneficial for relieving FD symptoms. Therefore, the present study was designed to evaluate the nutritional habits of FD patients, to examine the relationship between unhealthy nutritional habits and dyspeptic symptoms, and to determine the foods that may lead to postprandial fullness, early satiety, epigastric pain, and epigastric burning in patients with FD. According to the results of this study, we aimed to improve the quality of life of FD patients by recommending healthy dietary habits.

## Materials and Methods

### Study Design and Subjects

This is a descriptive cross-sectional study conducted in the endoscopy unit of a gastroenterology institute between March 2019 and August 2019. All patients who applied to the gastroenterology institute and were diagnosed with FD were contacted within these dates and 60 participants among 200 patients who were approved to participate in the study were included. The worldwide prevalence of FD is 5%-29%.^2,3^ When prevalence is chosen as 5%, the sample size is calculated as 54 patients with a 95% CI, 5% error. When prevalence is chosen as 20%, the sample size is calculated as 111 patients.

This study was approved by the ethics committee under the protocol number 09.2018.207. All participants signed the “informed consent form” and “consent form.”

The inclusion criteria of the study was patients within the age range of 18-65 years, absence of peptic ulcer, celiac disease, esophagitis, cancer, or any organic or metabolic disease that could explain dyspeptic symptoms, no dietary intervention that restricts energy and/or food intake, no previous abdominal or bariatric surgery, no psychiatric disease, not being in pregnancy or breastfeeding period, and not having been diagnosed with FD according to Rome IV criteria. Participants who did not meet these criteria were excluded from the study.

Participants must have 1 or more of the symptoms of postprandial fullness, early satiety, epigastric pain, and epigastric burning to comply with the Rome IV criteria, and the symptoms should have started at least 6 months before the diagnosis and have the diagnostic criteria for the last 3 months without organic diseases to explain these symptoms. Upper gastrointestinal system endoscopy should be normal.^1^

### Data Collection Tool

In the study, a questionnaire was applied to the participants by face-to-face interview method. The questionnaire form was prepared in consideration of the studies conducted by Filipovic et al.,^5^ Göktaş et al.,^6^ Hassanzadeh et al.,^11^ Jiang et al.,^12^ and Carvalho et al.^13^

In the questionnaire form, individuals’ demographic (age and gender) and anthropometric characteristics (height and body weight), nutritional habits (such as the number of snacks and main meals, duration and speed of eating, symptoms after meals, cooking styles, alcohol use, and smoking), and frequency of food consumption were included. The food frequency questionnaire (FFQ) is a global questionnaire applicable to all populations that assesses the frequency, portion sizes, and/or nutrient intake of foods and/or food groups over a period of time.^14^ Appropriate foods to be included in FFQ can be made based on past dietary surveys, beliefs, food choices, and literature. Accordingly, in our study, the frequency and amount of foods that could trigger FD symptoms were determined using FFQ.^5,6,11,12,13^

### Anthropometric Measurements

Participants’ body compositions were measured by TANITA MC 780 P brand scale using the bioelectrical impedance analysis (BIA) method. Body fat percentage, the amount of fat around the waist, and body mass index (BMI) of patients were determined with the BIA method. Participants were informed that they should be hungry for at least 3 hours before the measurement, should not be doing heavy physical activity 24-48 hours before the measurement, and women should not be in the menstrual period as it may affect the weighing result. Participants were measured with a seca mechanical height gauge without shoes, feet side by side, and head on the Frankfort horizontal plane. Body mass index was calculated using the participants’ height (m) and body weight (kg) values.

### Statistical Analysis

Statistical analysis was performed using the Statistical Package for Social Sciences version 20.0 software (IBM Corp.; Armonk, NY, USA). Descriptive statistics consisting of frequency, percentage, mean, and standard deviation (SD) were made for demographic variables. Chi-square and Mann–Whitney *U* tests were used to evaluate the difference in eating habits according to the frequency of FD symptoms. As a result of the analysis, *P* < .05 was considered statistically significant.

## Results

The average age of the participants was 43.78 ± 11.46 years and 71.7% (n = 43) of the participants were women. Their body weight and BMI were 73.84 ± 14.28 kg/m^2^ and 27.49 ± 5.28 kg/m^2^, respectively. Of the participants, 61.7% were overweight and their body fat percentage was on average 28.67 ± 8.58. Waist circumference, which is an indicator of abdominal obesity, was found to be an average of 10.21 ± 4.28 kg. Anthropometric measurements and general characteristics of the participants were shown in Table 1.

Eating habits, symptoms of FD, smoking, and alcohol use of patients are shown in Table 2. The majority of the participants consumed their meals within 10-20 minutes. While the proportion of those who consumed the breakfast meal for less than 10 minutes was 35%, the rate of those who consumed the evening meal between 10 and 20 minutes was determined as 48.3%. When asked to evaluate their chewing speed, 48.3% of them stated that they chewed fast and the rate of those who chew slowly was only 16.7%.

While 86.7% of the participants experienced postprandial fullness and 33.3% early satiety ≥1 day a week, 56.7% of them reported pain in the stomach and 68.3% of them reported burning in the stomach ≥1 day a week. The most common cooking method was roasting (81.7%; always) and boiling (53.3%; none) was the least common. Frying (46.7%) and baking (43.3%) methods were preferred occasionally. Of the participants, 95% ate breakfast and 91.7% ate dinner almost every day. It was observed that patients mostly preferred a snack at night (91.7%; 5-7 days a week). Most of the participants did not smoke or drink alcohol (63.3% and 71.7%, respectively).

The comparison of gender, alcohol use, and smoking according to FD symptoms are shown in Table 3. Stomach pain (*P* = .036) and heartburn (*P* = .026) experienced ≥1 day a week were more common in women than men. There was no difference in terms of gender in postprandial fullness and early satiety (*P* > .05).

According to the results obtained in this study, 28.3% of the participants with FD were alcohol consumers. Participants with stomach pain and heartburn symptoms of ≥1 day a week had consumed less alcohol (*P* = .007 and *P* = .026, respectively). No difference was found in the symptoms of postprandial fullness and early satiety (*P* > .05).

It was determined that the rate of participants who smoke was 36.7%. It was observed that participants who did not smoke have experienced early satiety symptoms <1 day a week (*P* = .037).

The difference in postprandial fullness symptoms in the stomach according to the cooking methods is shown in Table 4. It was found that the participants who always prefer the roasting method experienced postprandial fullness more frequently (*P* = .013). However, there was no difference between other symptoms and cooking methods (*P* > .05).

There was no statistically significant difference between FD symptoms and BMI and percentage of fat and abdominal fat (*P* > .05). When the frequency and duration of meals, and chewing speed of the participants were compared with the frequency of FD symptoms, no statistically significant difference was found (*P* > .05).

Fifty-one foods that can trigger symptoms in FD were questioned with a 1-month retrospective food consumption frequency questionnaire. A comparison of daily food consumption according to FD symptoms is shown in Table 5. The consumption of broccoli (*P* = .003), radish (*P* = .024), celery (*P* = .003), green olives (*P* = .013), and olive oil (*P* = .006) was observed less in those with increased postprandial fullness. Participants who had more pain in the stomach had less consumption of dried fruits (*P* = .017), green olives (*P* = .028), butter (*P* = .019), fast food (*P* = .032), and alcohol (*P* = .031) but the consumption of sunflower oil (*P* = .022) was higher. It was found that the participants who had more burning symptoms in the stomach had less alcohol (*P* = .011) and fast food (*P* = .023) consumption.

## Discussion

This study was conducted to examine the nutritional habits of patients with a diagnosis of FD. In our study, which included 60 patients with a diagnosis of FD, it was observed that the average age of the participants was 43.78 ± 11.46 years and 71.7% of them were women. When the symptoms of FD and the gender of the individuals were compared, it was found that the symptoms of stomach pain and burning in the stomach were more common in women. Several epidemiological studies have shown that the prevalence of FD was higher in women.^15-17^ Kawakubo et al.^17^ examined upper gastrointestinal symptoms in healthy young people and found that the symptoms of postprandial fullness, stomach pain, unintentional chest rubbing, and early satiety were higher in women. It was found that 60% of the adult patients diagnosed with FD according to the Rome IV criteria in England, the United States of America, and Canada were female.^15^ A study in Japan reported that the prevalence of FD was 26% in women and 11% in men.^17^ While ulcer-like dyspepsia is more common in men (73.3%), dysmotility-like dyspepsia is more frequent in women (57%). It has been reported that the relationship between gender and FD is not statistically significant.^5^

It is thought that there is a relationship between increased BMI and the prevalence of functional gastrointestinal disease.^18^ Our findings were different from other studies. In our study, 61.7% of the participants were overweight and their BMI values were found to be 27.49 ± 5.28 kg/m^2^ on average. Body fat percentages were on average 28.67 ± 8.58%, while abdominal fat amounts were 10.21 ± 4.28 kg on average. However, although most of the participants in our study were slightly overweight or obese, no significant difference was found between FD symptoms and BMI, fat percentage, and waist circumference. In a recent study, it was found that the relationship between FD and BMI changes according to gender. It has been reported that FD is observed at a statistically significant higher rate in slightly overweight and obese women.^19^ High BMI was found to be an independent risk factor in dyspepsia occurrence at a 10-year follow-up in an England cohort study.^20^ In addition to BMI, visceral fat should also be considered as an important risk factor in FD.^21^

Chewing speed plays an important role in triggering FD symptoms.^22^ When asked to evaluate their chewing speed in our study, 48.3% of participants stated that their chewing speed is fast and only 16.7% indicated that their chewing speed is slow. Participants’ eating duration was usually between 10 and 20 minutes. Although most of the patients participating in our study stated that they ate fast, there was no statistically significant difference between FD symptoms and chewing speed and eating duration. Similar to our finding, Sinn et al.^23^ reported that participants with FD reported higher chewing speed than healthy individuals; but when real-time eating times were measured, it was determined that this difference was not significant.^23^ However, 1 study found that the reduced risk of FD may be due to extended lunch time. This relationship is explained by the postprandial fullness and stomach discomfort that may occur as a result of an inability to swallow properly due to fast food consumption.^22^ Although no interventional studies have been reported so far, patients may be advised to eat slowly and regularly, especially in the subgroup of patients with delayed gastric emptying.^24^

It has been suggested that there is a relationship between meal frequency, irregular eating and skipping meals, and symptoms of FD.^11^ In our study, although there was no significant difference between FD symptoms and irregular eating and skipping meals, most of the participants reported that they ate irregular meals and skipped meals. In a cross-sectional study conducted in Iran, it was found that those who consumed 3 main meals a day experienced 52% fewer FD symptoms than those who consumed 1 main meal, and an inverse relationship was found between the number of meals and FD.^11^ Xu et al.^4^ reported that irregular eating duration is a risk factor for upper abdominal pain. On the other hand, it was found that participants with FD skip breakfast more often, but this situation was not statistically significant on the elevated risk of FD.^22^ Another study in Turkey also found no significant relationship between the main meal and snack frequency with the FD.^6^ The consumption of small and frequent meals may be a reasonable recommendation to reduce the symptoms of FD.^25^

In our study, cooking methods were questioned under 4 headings: boiling, roasting, frying, and baking. According to our results, participants preferred roasting the most and boiling the least. When the cooking methods and FD symptoms were compared, it was found that the participants with more postprandial fullness in the stomach always preferred the roasting method. The oil used for roasting and frying is oxidized into hydroperoxides at high temperatures in the presence of oxygen. Acrolein, which is formed as a result of oxidation, is a pungent substance that irritates the mouth and nasal mucosa and creates bitterness in the taste of the oil.^26^ It is thought that this change in fat can trigger postprandial fullness in the stomach. Participants in our study preferred roasting as a cooking method, which may be due to their ongoing habits and ignoring the increase in postprandial fullness symptoms. There is no similar study examining this comparison in the literature.

While smoking increases gastric acid secretion and pepsinogen release, it may delay gastric emptying and cause gastrointestinal disturbances.^27^ Patients diagnosed with FD according to endoscopy results and Rome IV criteria were included in our study, and 36.7% of the participants stated that they smoke. It was determined that patients who experienced fewer symptoms of early satiety did not smoke. Smoking has been identified as a risk factor that accelerates the pathogenesis of FD during the 10-year period.^15^ However, it has also been shown that there is no relationship between FD and smoking in patients with FD diagnosed according to Rome III criteria.^28^ Despite there being a strong relationship between FD and smoking, which is revealed by several epidemiological studies and initial surveys in the population, studies related to gastrointestinal system endoscopy, Roma criteria, and complete medical history showed a slight relationship.^29^ In addition, it is thought that smoking does not trigger FD symptoms because it is effective in reducing appetite and decreasing food consumption.^30^

Many different studies have shown that alcohol consumption is not associated with FD.^16,28^ In our study, it was found that the participants who experienced stomach pain and burning symptoms for ≥1 day a week had less alcohol consumption. These patients may have preferred less alcohol consumption because they thought that alcohol would trigger FD symptoms. Halder et al.^24^ found a relationship between increased alcohol consumption (>6 cups/week) and the presence of FD.^31^ While alcohol consumed in large amounts affects FD, moderate alcohol intake does not affect the presence or extent of dyspeptic symptoms.^24^

A potential association between gastrointestinal symptoms and certain foods has been reported in different studies. Accordingly, it was observed that the consumption of broccoli, radish, celery, green olives, and olive oil decreased in the participants who experienced more postprandial fullness symptoms. It was found that the participants with stomach pain symptoms ≥1 day a week consumed more sunflower oil. While the consumption of dried fruits, green olives, butter, alcohol, and fast food was less in those with severe stomach pain, alcohol and fast food consumption was found to be less in those with severe stomach burning. Participants may prefer to avoid or consume less certain foods that are known to trigger FD symptoms, such as celery, broccoli, radish, and fast food, which may explain our findings. While spicy and fatty foods, and sweet snacks are considered as risk factors in FD, the relationship between tea and coffee consumption and FD is controversial.^6,12^ In one of the studies, it was observed that dyspeptic symptoms increased in 44% of the FD patients after milk consumption.^13^ Göktaş et al.^6^ showed that foods that trigger the symptoms in all subgroups of FD are fried and fatty foods, hot spices, carbonated drinks, dried legumes, bulgur, rice, onion, leek, and green onion. It has also been shown that high fruit consumption is related to lower odds of early satiation and postprandial fullness. Additionally, higher intake of vegetables is associated with lower risk of FD symptoms only in men.^32^

There are limitations of our study, such as the small sample size, 1-time food consumption record, and the inability to direct more detailed questions to the participants about the foods consumed due to time restrictions.

## Conclusion

There is no similar study in the literature examining the difference between cooking style and FD symptoms. In our study, it was found that those who always prefer roasting as a cooking method experienced postprandial fullness ≥1 day a week. Another finding of our study is that early satiety is less common in non-smokers.

In conclusion, in our study, it was observed that patients with FD either intentionally or unintentionally avoided some foods that may trigger symptoms or consumed less of these foods. Reducing the consumption of foods such as broccoli, radish, celery, green olives, olive oil, dry fruits, and butter that trigger FD symptoms, reducing smoking and consumption of alcohol and fast food, and avoiding unhealthy cooking methods such as roasting are suggested to relieve the symptoms and to improve the quality of life of FD patients. It is recommended that patients with FD should be informed in detail by a dietician in order to be aware of the eating habits that may exacerbate their symptoms. In addition, different studies are needed to reveal the relationship between FD and nutritional status with a larger sample.

## Figures and Tables

**Table 1.** Descriptive Characteristics of the Participants

Variables	Total
Anthropometric characteristics mean ± SD (min–max)	
Age (years)	43.78 ± 11.46 (19.00-65.00)
Height (cm)	164.01 ± 8.06 (146.00-185.00)
Weight (kg)	73.84 ± 14.28 (48.30-119.50)
Body mass index (kg/m^2^)	27.49 ± 5.28 (17.74-46.10)
Percentage of body fat (%)	28.67 ± 8.58 (9.30-43.40)
Fat around the waist (kg)	10.21 ± 4.28 (0.80-22.00)
Gender, n (%)	
Female	43 (71.7)
Male	17 (28.3)
Body mass index (kg/m^2^), n (%)	
Normal (18.5-24.9 kg/m^2^)	23 (38.3)
Overweight (>25.0 kg/m^2^)	37 (61.7)

SD, standard deviation.

**Table 2.** Nutritional Habits of the Participants

Variables	n (%)
Main meals	
Breakfast	
5-7 days a week	57 (95.0)
≤4 days a week	3 (5.0)
Lunch	
5-7 days a week	27 (45.0)
≤4 days a week	33 (55.0)
Dinner	
5-7 days a week	55 (91.7)
≤4 days a week	5 (8.3)
Snacks	
Mid-morning	
5-7 days a week	12 (20.0)
≤4 days a week	48 (80.0)
Afternoon	
5-7 days a week	27 (45.0)
≤4 days a week	33 (55.0)
Night	
5-7 days a week	55 (91.7)
≤4 days a week	5 (8.3)
Eating duration	
Breakfast	
None	0 (0)
<10 minutes	21 (35.0)
10-20 minutes	22 (36.7)
>20 minutes	17 (28.3)
Lunch	
None	21 (35.0)
<10 minutes	11 (18.3)
10-20 minutes	23 (38.3)
>20 minutes	5 (8.3)
Dinner	
None	1 (1.7)
<10 minutes	14 (23.3)
10-20 minutes	29 (48.3)
>20 minutes	16 (26.7)
Chewing speed	
Slow	10 (16.7)
Normal	21 (35.0)
Fast	29 (48.3)
Functional dyspepsia symptoms	
Postprandial fullness	
<1 day a week	8 (13.3)
≥1 days a week	52 (86.7)
Early satiety	
<1 day a week	40 (66.7)
≥1 days a week	20 (33.3)
Pain in the stomach	
<1 day a week	26 (43.3)
≥1 days a week	34 (56.7)
Burning in the stomach	
<1 day a week	19 (31.7)
≥1 days a week	41 (68.3)
Cooking methods	
Boiling	
No	32 (53.3)
Sometimes	19 (31.7)
Often	9 (15.0)
Roasting	
Often	11 (18.3)
Always	49 (81.7)
Frying	
No	10 (16.7)
Sometimes	28 (46.7)
Often	22 (36.7)
Baking	
No	13 (21.7)
Sometimes	26 (43.3)
Often	21 (35.0)
Alcohol use	
No	43 (71.7)
Yes	17 (28.3)
Smoking	
No	38 (63.3)
Yes	22 (36.7)

**Table 3.** Comparison of Gender, Alcohol Use, and Smoking According to FD Symptoms

	<1 Day a Week	≥1 Day a Week	χ^2^	*P*
Gender				
Postprandial fullness				
Female (n = 43)	5	38	0.382	.676
Male (n = 17)	3	14
**Early satiety**				
Female	27	16	1.026	.311
Male	13	4
**Pain in the stomach**				
Female	15	28	4.413	**.036**
Male	11	6
**Burning in the stomach**				
Female	10	33	4.962	**.026**
Male	9	8
Alcohol use				
**Postprandial fullness**				
No (n = 43)	5	38	0.382	.676
Yes (n = 17)	3	14
**Early satiety**				
No	29	14	0.041	.839
Yes	11	6
**Pain in the stomach**				
No	14	29	7.176	**.007**
Yes	12	5
**Burning in the stomach**				
No	10	33	4.962	**.026**
Yes	9	8
Smoking				
Postprandial Fullness				
No (n = 38)	7	31	2.321	.238
Yes (n = 22)	1	21
**Early satiety**				
No	29	9	4.342	**.037**
Yes	11	11
**Pain in the stomach**				
No	14	24	1.778	.182
Yes	12	10
**Burning in the stomach**				
No	13	25	0.310	.578
Yes	6	16

FD, functional dyspepsia; *P* < .05; chi-squared test. The significance of bold values P <.05.

**Table 4.** Comparison of Cooking Methods According to the Symptom of Postprandial Fullness in the Stomach

Cooking Methods	Frequency	Postprandial Fullness		Total	χ^2^	*P*
<1 Day a Week	≥1 Day a Week
Boiling	No	4	28	32	0.157	.925
	Sometimes	3	16	19		
Often	1	8	9
Roasting	Often	4	7	11	6.182	**.013**
Always	4	45	49
Baking	No	1	12	13	1.404	.496
	Sometimes	5	21	26		
Often	2	19	21
Frying	No	3	7	10	3.896	.143
	Sometimes	4	24	28		
Often	1	21	22

*The significance of bold valuesP* < .05; chi-squared test.

**Table 5.** Comparison of Daily Food Consumption According to FD Symptoms

Foods (g)	<1 Day a WeekMedian (Q1-Q3)	≥1 Day a WeekMedian (Q1-Q3)	Z	*P*
Postprandial Fullness	(n = 8; 13.3%)	(n = 52; 86.7%)		
Broccoli	20.0 (0.5-30.0)	0 (0-3.3)	−2.944	.003
Radish	9.0 (0.3-20.8)	0 (0-4.8)	−2.259	.024
Celery	5.0 (0-17.5)	0 (0-0)	−2.993	.003
Green olives	7.0 (0.8-13.8)	0 (0-4.8)	−2.487	.013
Olive oil (mL)	25.0 (11.0-30.0)	10.0 (4.3-20.0)	−2.729	.006
**Pain in the stomach**	**(n = 26; 43.3%)**	**(n = 34; 56.7%)**		
Dried fruits	4.5 (0.6-16.3)	0.3 (0-4.0)	−2.391	.017
Green olives	0.2 (0-10.0)	0 (0-3.3)	−2.193	.028
Sunflower oil (mL)	4.0 (0.3-10.0)	10.0 (2.0-21.3)	−2.284	.022
Butter	5.0 (1.9-10.0)	2.5 (0.9-5.0)	−2.337	.019
Fast food	0 (0-47.7)	0 (0-1.1)	−2.148	.032
Alcohol (mL)	0 (0-10.8)	0 (0-0)	−2.153	.031
**Burning in the stomach**	**(n = 19; 31.7%)**	**(n = 41; 68.3%)**		
Fast food	11.6 (0-40.2)	0 (0-2.1)	−2.269	.023
Alcohol (mL)	0 (0-13.4)	0 (0-0)	−2.547	.011

FD, functional dyspepsia; *P* < .05; Mann–Whitney *U* test.
